# The Lack of WIP Binding to Actin Results in Impaired B Cell Migration and Altered Humoral Immune Responses

**DOI:** 10.1016/j.celrep.2018.06.051

**Published:** 2018-07-17

**Authors:** Selina Jessica Keppler, Marianne Burbage, Francesca Gasparrini, Lara Hartjes, Shweta Aggarwal, Michel J. Massaad, Raif S. Geha, Andreas Bruckbauer, Facundo D. Batista

**Affiliations:** 1 Lymphocyte Interaction Laboratory, Francis Crick Institute, London NW1 1AT, UK; 2 Division of Immunology, Children’s Hospital and Department of Pediatrics, Harvard Medical School, Boston, MA 02115, USA; 3 Ragon Institute of MGH, MIT and Harvard, Cambridge, MA 02139, USA; 4 MRI, TranslaTUM, Institute for Clinical Chemistry and Pathobiochemistry, Immune Signals and Cancer, 81675 Munich, Germany

**Keywords:** B lymphocytes, chemotaxis, actin cytoskeleton, CXCR4, HS1, Wiskott-Aldrich syndrome, PI3K signaling

## Abstract

Wiskott-Aldrich syndrome protein (WASp) is a main cytoskeletal regulator in B cells. WASp-interacting protein (WIP) binds to and stabilizes WASp but also interacts with actin. Using mice with a mutated actin binding domain of WIP (WIPΔABD), we here investigated the role of WIP binding to actin during B cell activation. We found an altered differentiation of WIPΔABD B cells and diminished antibody affinity maturation after immunization. Mechanistically, WIPΔABD B cells showed impaired B cell receptor (BCR)-induced PI3K signaling and actin reorganization, likely caused by diminished CD81 expression and altered CD19 dynamics on the B cell surface. WIPΔABD B cells displayed reduced *in vivo* motility, concomitantly with impaired chemotaxis and defective F-actin polarization, HS1 phosphorylation, and polarization of HS1 to F-actin-rich structures after CXCL12 stimulation *in vitro*. We thus concluded that WIP binding to actin, independent of its binding to WASp, is critical for actin cytoskeleton plasticity in B cells.

## Highlights

• Binding of WIP to actin influences CD19 phosphorylation and PI3K signaling
• Actin reorganization and HS1 localization are disturbed in WIPΔABD mutant B cells
• Faulty actin plasticity results in reduced B cell chemotaxis and migration
• Binding of WIP to actin in B cells shapes humoral immune responses

## Graphical Abstract

**Figure:**
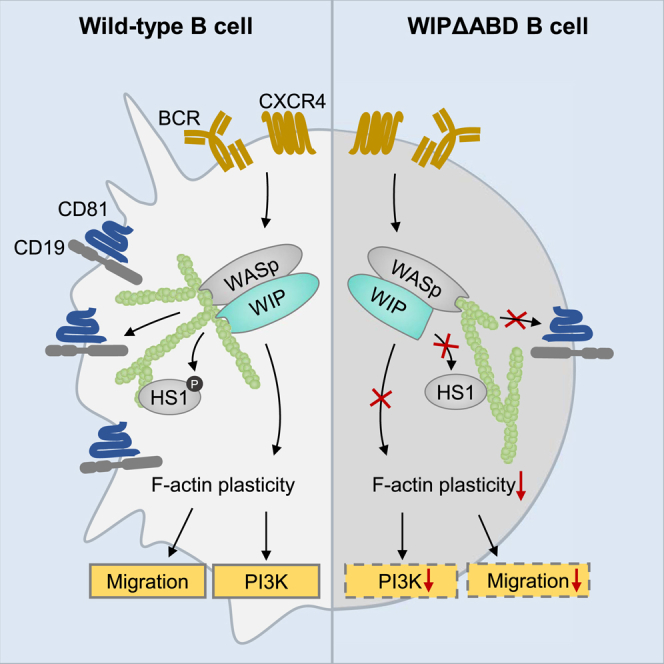

## Teaser

The absence of WIP in mouse and human triggers immunodeficiency mimicking Wiskott-Aldrich syndrome. Keppler et al. report that binding of WIP to actin shapes actin cytoskeleton plasticity in B cells; is sufficient to modulate signaling, chemotaxis, and *in vivo* migration; and hence influences humoral immune responses.

## Introduction

B cells are an integral part of the adaptive immune system. Although correct activation of B cells protects the host against a variety of infections and cancers, aberrant B cell activation has been implicated in the development of autoimmune diseases. The actin cytoskeleton plays a central role in regulating this lymphocyte activation. In steady state, the B cell receptor (BCR) is immobilized on the plasma membrane by areas of high-density cortical actin cytoskeleton. Antigen-induced BCR signaling leads to an early rapid wave of actin depolymerization (Hao and August, 2005), removing barriers to BCR diffusion and modifying BCR dynamics at the cell surface. Furthermore, changes in actin organization alone increase BCR diffusion in a ligand-independent manner and trigger signaling similar to BCR crosslinking (Treanor et al., 2010), involving the co-receptor CD19 held in place by its association with the tetraspanin molecule CD81 (Mattila et al., 2013). Hence, the actin cytoskeleton organizes and immobilizes the BCR and regulates the BCR proximity to its co-receptors in the plasma membrane (Gasparrini et al., 2016, Mattila et al., 2016, Song et al., 2014, Tolar, 2017) but is also needed for the correct reorganization of the plasma membrane surface in response to extracellular clues, thereby regulating intracellular signaling.

Regulators of actin dynamics that limit BCR mobility and thus antigen-independent BCR signaling may prevent spontaneous B cell activation. As a consequence, lack of these actin regulators can result in disease, as seen in Wiskott-Aldrich syndrome (WAS). WAS is an X-linked disorder associated with eczema, increased susceptibility to infections, and heightened risk of autoimmune disorders (Thrasher, 2009), which is caused by mutations in Wiskott-Aldrich syndrome protein (WASp) (Stewart et al., 1999) or in WASp-interacting protein (WIP) (Lanzi et al., 2012, Pfajfer et al., 2017). Functional defects are more severe in B cells of *Wipf1*^*−*^^*/*^^*−*^ mice, which lack WIP and WASp, than in B cells of WASp-deficient mice, suggesting that WIP interaction with actin might be crucial for B cell cytoskeletal plasticity and function. WIP binding to WASp protects it from degradation and regulates its cellular distribution (Fried et al., 2014). However, WIP promotes actin polymerization independently of WASp by binding and stabilizing actin filaments (Martinez-Quiles et al., 2001, Ramesh et al., 1997). Binding of WIP to actin is mediated by the N-terminal verprolin homology region that includes an amino acid sequence (amino acids 43–54) containing a KLKK motif critical for actin binding to thymosin b4 (Antón et al., 2003, Van Troys et al., 1996). Using *Wipf1*^*−*^^*/*^^*−*^ mice, we have shown that WIP regulates B cell homing, chemotaxis, survival, and differentiation due to an altered CD19 cell surface dynamics, resulting in impaired phosphatidylinositol 3-kinase (PI3K) signaling after triggering a variety of receptors (Keppler et al., 2015). However, the role of WIP binding to actin, in contrast to its WASp stabilizing function in B cells, has not been studied so far. Mice lacking the actin binding domain (ABD) of WIP (WIPΔABD) have been generated (Massaad et al., 2014), and T cells of these mice displayed decreased cellular filamentous actin (F-actin) content, impaired chemotaxis, and defective homing to lymph nodes despite having normal WASp expression (Gallego et al., 2006, Massaad et al., 2014). Here, we dissected the role of WIP binding to actin from its WASp stabilizing function during B cell activation.

We found that the lack of WIP binding to actin in B cells resulted in an altered humoral immune response with reduced antibody affinity maturation in response to immunization. We furthermore demonstrated that the binding of WIP to actin alone influences CD81 expression and hence CD19 diffusion on the B cell surface, which correlated with an impaired actin cytoskeletal reorganization and diminished PI3K signaling after BCR and CXCR4 stimulation. The binding of WIP to actin is sufficient to regulate B cell chemotaxis to CXCL12 and *in vivo* migration. On a more molecular level, we found a defective F-actin polarization, together with a diminished localization of HS1 in F-actin rich structures, after CXCL12 stimulation of B cells lacking the binding of WIP to actin. From these results, we concluded that the binding of WIP to actin, independent of its binding to WASp, is critical for actin cytoskeleton plasticity in B cells, thereby influencing PI3K signaling, migration, and antibody production.

## Results

### B Cells Lacking the Binding of WIP to Actin Demonstrate Altered Humoral Immune Responses

**Figure 1:**
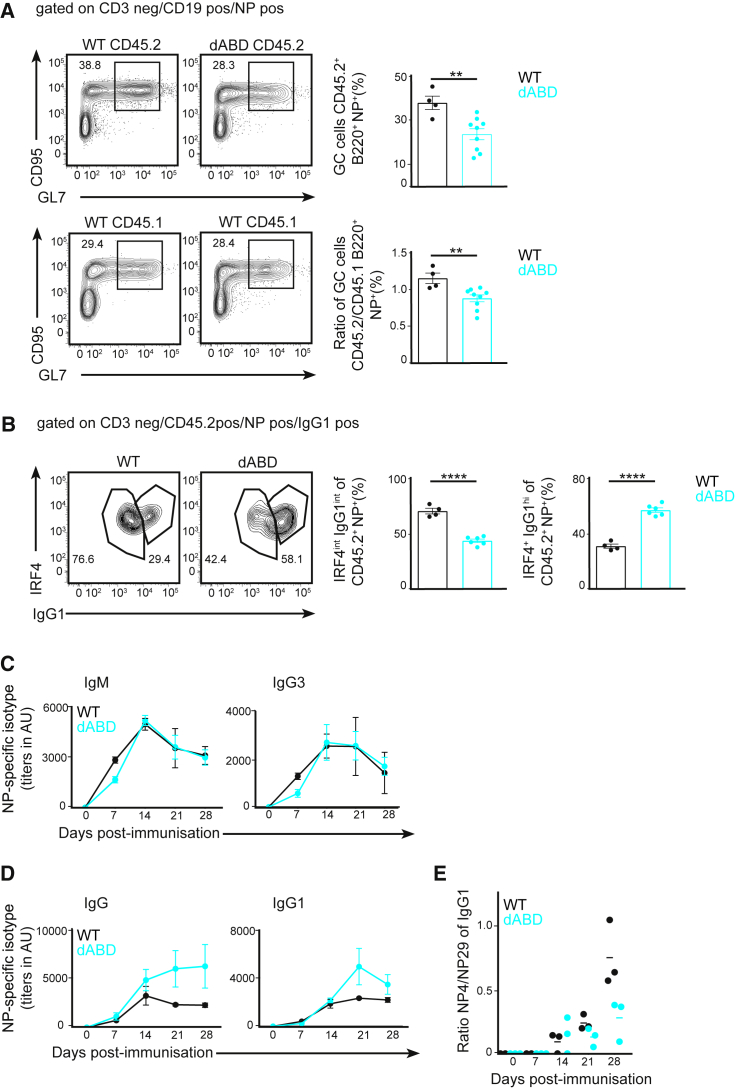
Compromised Germinal Center Formation and Altered Antibody Affinity Maturation in B Cells Lacking Binding of WIP to Actin (A and B) Lethally irradiated CD45.1 recipients were reconstituted for 10 weeks with mixtures of 50% WT CD45.1^+^ BM and 50% WT or WIPΔABD CD45.2^+^ BM, immunized with NP-KLH in alum and spleens of chimeras analyzed by flow cytometry at day 13. Data (mean ± SEM) are representative of two independent experiments with at least 4 mice per group. (A) CD45.1^+^ and CD45.2^+^ NP-specific GC B cells (B220^+^, NP^+^, GL7^+^, and CD95^+^) are shown. Graphs on the right indicate the percentages of CD45.2^+^ GC cells and the CD45.2/CD45.1 ratio of GC B cells. (B) CD45.2^+^, NP-specific, and IgG1^+^ cells expressing the transcription factor IRF4 are shown. Graphs on the right indicate the percentages of IRF4^int^IgG1^int^ and IRF4^+^IgG1^hi^ cells. (C–E) JHT-WT or JHT-WIPΔABD mixed BM chimeras were immunized with NP-KLH in alum. NP_29_-specific IgM and IgG3 (C) and IgG and IgG1 (D) antibodies in the sera of immunized chimeras were determined at the indicated time points by ELISA. Affinity maturation (expressed as the ratio of NP_4_ to NP_29_ ELISA titers of IgG1 antibodies) is shown in (E). Data (mean ± SEM) are representative of two independent experiments with at least 3 mice per group. See also Figure S1.

We previously showed that the absence of WIP exclusively in B cells impairs mouse immune responses by compromising germinal center (GC) responses and antibody production (Keppler et al., 2015). To establish whether WIP binding to actin has an effect on humoral immune responses, we generated mixed bone marrow (BM) chimeras by reconstituting lethally irradiated congenic BALB/c CD45.1 animals with a mixture of 50% CD45.1 wild-type (WT) BM and 50% CD45.2 WIPΔABD mutant BM (WT-WIPΔABD), WIP-deficient BM (WT-*Wipf1*^−/−^), or WT-WT BM. These mixed chimeras were used to analyze reconstitution and the formation of GC B cells in a competitive environment. During analysis of these mixed chimeras, we found a 50% reduction of CD45.2 *Wipf1*^−/−^ B cells compared to CD45.1 WT B cells, altering the ratio of CD45.1 to CD45.2 B cells from 50:50 initially to 80:20 in the spleen, similar to what we have described previously (Figure S1A) (Keppler et al., 2015). We further detected a 20% reduction of CD45.2 WIPΔABD B cells compared to CD45.1 WT B cells (Figure S1A), suggesting that under these conditions, WT cells also partially outcompete WIPΔABD B cells for access to the mature B cell niches. Next, chimeric mice were challenged with the T cell-dependent antigen (4-Hydroxy-3-Nitrophenyl)Acetyl - Keyhole Limpet Hemocyanin (NP-KLH) in alum and analyzed 13 days later using flow cytometry. We observed a robust formation of GC B cells in the spleens of all immunized chimeras, with about 29% of CD45.1 WT B cell staining positive for GC markers (GL7^+^CD95^+^NP^+^ of B220^+^) (Figure 1A, lower panel). We used the percentages of CD45.2^+^ GC cells, as well as the ratio of CD45.2/CD45.1 GC B cells, as a readout to compare the ability of WT, WIPΔABD, and *Wipf1*^*−/−*^ B cells to compete with the CD45.1 WT cells present in the same animal. We found that, similar to CD45.2 *Wipf1*^−/−^ B cells (Figure S1B), CD45.2 WIPΔABD B cells were significantly impaired in GC formation compared to CD45.2 WT B cells (Figure 1A, upper panel and graphs on the right).

Next, we analyzed class-switching to immunoglobulin (Ig) G1 and expression of interferon regulated factor 4 (IRF4) —a hallmark transcription factor for plasma cell differentiation. During the GC response, IRF4 downregulation is required for the B cells to stay in the GC, whereas cells expressing high levels of IRF4 exit the GC to become plasma cells (Li et al., 2018). Intracellular staining of NP-specific CD45.2 B cells revealed two populations: one expressing IRF4 and intermediate levels of IgG1 (IRF4^int^ and IgG1^int^) and a population expressing high levels of IgG1 and further upregulation of IRF4 (IRF4^+^ and IgG1^hi^). A higher percentage of WIPΔABD B cells differentiated into IRF4^+^- and IgG1^hi^-expressing cells (58% of CD45.2 cells) compared to WT or *Wipf1*^−/−^ B cells (29.4% or 27% of CD45.2 cells, respectively) (Figure 1B; Figure S1C). These results suggest a preferential engagement of WIPΔABD B cells into the plasma cell differentiation pathway.

To analyze antibody responses after immunization, we next generated mixed BM chimeras by adoptively transferring a mix of BM from either WT or WIPΔABD mice and BM from mice that lack B cells (JHT mice) into lethally irradiated JHT recipients. BM of both WT and WIPΔABD mice reconstituted the B cell compartment in the spleen, but BM from WIPΔABD mice showed reduced reconstitution in the BM of recipient mice (Figure S1D). Purified splenic B cells of WIPΔABD mixed BM chimeras demonstrated detectable WASp levels in immunoblot, indicating that WASp stabilization is intact in B cells carrying the mutant WIP gene locus (Figure S1E). B cell population analysis in the spleen of WIPΔABD chimeras by flow cytometry showed similar marginal zone (MZ) populations (CD21^hi^ and CD23^int^) and mature B cell populations (IgD^hi^ and IgM^int^) when compared to WT chimeras, unlike B cells from *Wipf1*^−/−^ chimeras, which lack MZ B cells (Figure S1F).

Following reconstitution, chimeric mice were challenged with the T cell-dependent antigen NP-KLH in alum. Quantifying early NP-specific antibody titers in the serum of immunized mice by ELISA, we found that WIPΔABD chimeras had similar yet slightly delayed NP-specific IgM and IgG3 antibody concentrations compared to WT chimeras (Figure 1C). The production of NP-specific class-switched antibodies was increased at later time points in WIPΔABD chimeras, with a 2-fold increase of total IgG antibodies and a slight increase of IgG1 antibodies compared to WT chimeras (Figure 1D). To check for affinity maturation of the class-switched antibodies, we compared the ratio of the IgG1 binding efficiency to NP_4_ (higher affinity) and NP_29_ (lower affinity). We observed a steady increase in the NP_4_/NP_29_ ratio of WT antibodies, indicating the production of high-affinity antibodies over time. The NP_4_/NP_29_ ratio in the sera of WIPΔABD chimeras was about 2-fold lower at day 28 after immunization (Figure 1E). Using flow cytometry, the generation of GC B cells was measured 13 days after immunization in the spleen of chimeric mice. We detected a robust formation of GC (B220^+^, NP^+^, GL7^+^, and CD95^+^) B cells in both JHT-WT and JHT-WIPΔABD chimeras (Figure S1G), as reflected in numbers of GC-positive cells per spleen. These results indicate that although the GC formation and titers of NP-specific class-switched antibodies are comparable or even higher in sera of WIPΔABD compared to WT chimeras, these antibodies fail to affinity mature after immunization.

Collectively, these *in vivo* experiments suggest that B cells lacking the ABD of WIP have an altered humoral immune response to immunization, with hampered antibody affinity maturation and, in a competitive environment, defective GC formation.

### Binding of WIP to Actin Influences PI3K Signaling and BCR-Induced Actin Reorganization

**Figure 2:**
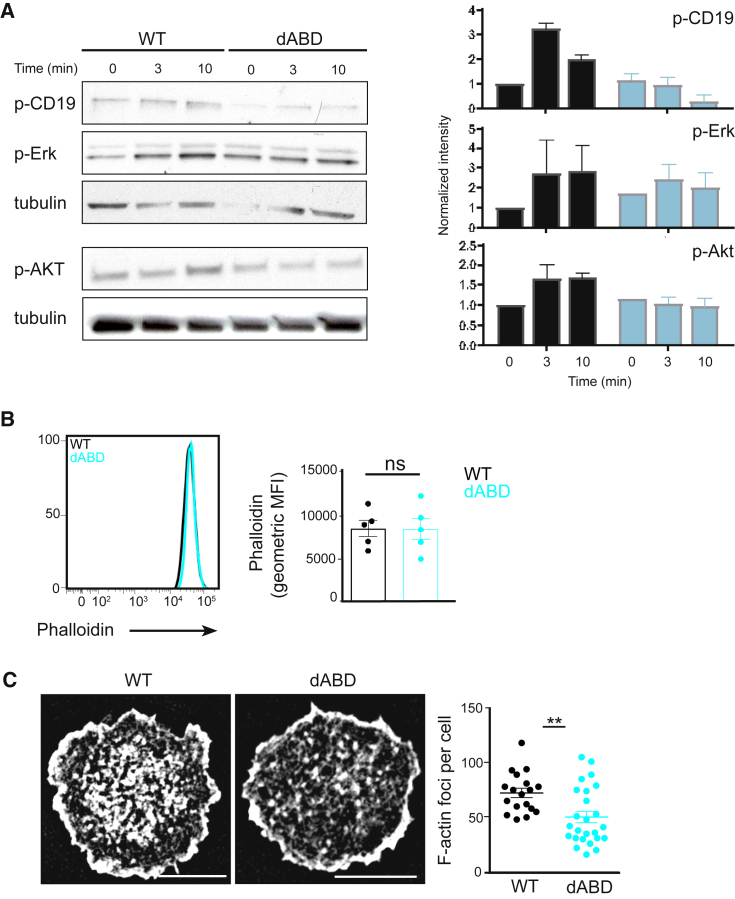
WIP Binding to Actin Influences PI3K Signaling and BCR-Induced Actin Reorganization (A) Immunoblot of splenic WT or WIPΔABD B cells stimulated with immobilized anti-κ-chain antibody on beads and probed with antibodies as indicated. Quantifications of intensity of proteins normalized by densitometry to tubulin and to the signal in unstimulated WT cells at t = 0 on the right. This experiment and the experiment in Figure S3F were performed simultaneously. The loading control measurements were part of both experiments. Data (mean ± SEM) are representative of at least two independent experiments. (B) Flow cytometric analysis of intracellular phalloidin of WT and WIPΔABD B cells. Quantification of the geometric mean fluorescence intensity (gMFI) is shown on the right (mean ± SEM). (C) Representative SIM images of phalloidin staining indicating the intracellular amount of F-actin in fixed WT or WIPΔABD B cells settled on coverslips coated with anti-κ-chain antibody for 10 min. Scale bar, 4 μm. Quantification (right graph) shows F-actin foci per cell (mean ± SEM). Data are representative of three independent experiments. See also Figure S2.

To account for the altered differentiation of WIPΔABD mutant B cells after immunization, we analyzed the influence of WIP binding to actin on BCR signaling. For this, we stimulated WT and WIPΔABD B cells with immobilized anti-κ-chain antibody on beads and measured the BCR-induced phosphorylation of the co-receptor CD19 and the kinases Akt and Erk by immunoblot. Both cell types displayed robust phosphorylation of Erk after BCR crosslinking (Figure 2A). However, the phosphorylation of CD19 as well as Akt was not detectable in WIPΔABD mutant B cells compared to WT cells (Figure 2A). We also obtained a reduced phosphorylation of CD19 and Akt in WIPΔABD B cells compared to WT cells using stimulation with soluble anti-κ-chain antibody (Figure S2A).

To determine whether this diminished signaling after BCR stimulation might be caused by altered actin regulation, we next analyzed levels of F-actin in steady-state WT and WIPΔABD B cells. Phalloidin staining revealed comparable total amounts of F-actin in naive WIPΔABD B cells and WT B cells (Figure 2B), as measured by flow cytometry. The total increase in F-actin per cell after soluble BCR activation was impaired in WIPΔABD B cells compared to WT cells (Figure S2B). Next, we investigated whether WIP binding to actin affects the reorganization of F-actin in activated B cells. For this, WT and WIPΔABD B cells were spread on coverslips with immobilized antigen, stained for F-actin with phalloidin and analyzed using structured illumination microscopy (SIM) imaging. Although WT B cells exhibited a characteristic peripheral F-actin ring and about 70 F-actin foci per cell, WIPΔABD B cells exhibited a significantly lower amount of F-actin foci with an average of 50 foci per cell (Figure 2C). The defects in F-actin formation resulted neither in the incapacity to spread nor in a defect of F-actin polymerization at the leading edge of WIPΔABD. Altogether, these results point at a reduced plasticity of the actin cytoskeleton after stimulation in B cells lacking WIP binding to actin.

Collectively, these results suggest that the binding of WIP to actin influences BCR-induced actin reorganization and is implicated in BCR-driven PI3K signaling likely influencing the differentiation of B cells after activation.

### Binding of WIP to Actin Affects CD19 Diffusion on the B Cell Surface

**Figure 3:**
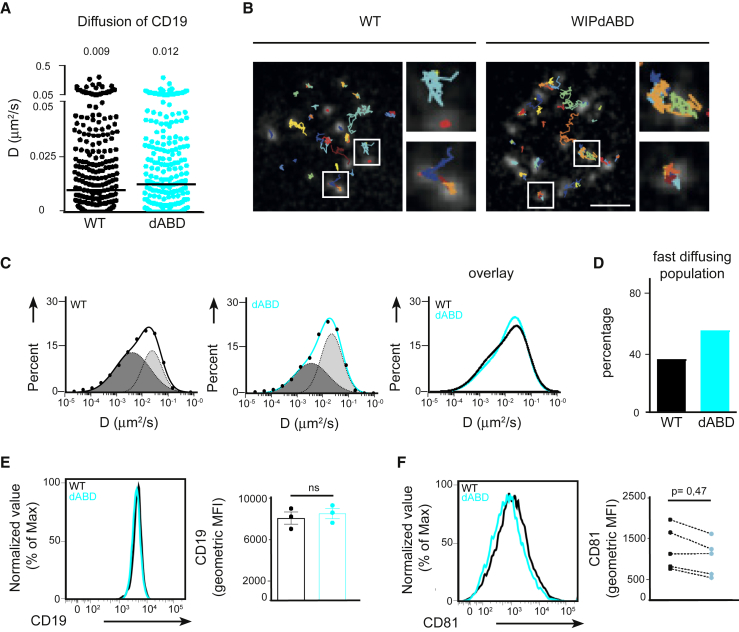
WIP Binding to Actin Influences CD19 Mobility and CD81 Expression on the B Cell Surface (A) Single-particle tracking (SPT) of CD19 on WT and WIPΔABD B cells settled on nonstimulatory coverslips. Shown is the diffusion coefficient (D) in square micrometers per second. The median of 300 representative tracks is given as a value and indicated by black bars. 1,000–2,000 tracks were analyzed with a minimum of 20 cells from 2 experiments. (B) Trajectories of CD19 in WT (left) and WIPΔABD B cells (right) showing diffusion of single particles over 10 s and magnified regions from white rectangles. Scale bar, 2 μm. (C) Analysis of the CD19 diffusion coefficient plotted in a logarithmic histogram and fitted to two distinct populations exhibiting slower (dark gray) and faster (light gray) diffusion on WT and WIPΔABD B cells. (D) The percentage of the fast-diffusing population calculated from the histograms. (E and F) Flow cytometric analysis of CD19 (E) and CD81 (F) on the surface of WT and WIPΔABD B cells. Quantification shows the gMFI (mean ± SEM). See also Video S1.

We previously showed that in the absence of WIP, the actin cytoskeleton, as well as the CD81 tetraspanin network, is disturbed, leading to increased diffusion of CD19 on resting B cells and resulting in impaired PI3K signaling (Keppler et al., 2015). Because we observed reduced CD19, as well as Akt phosphorylation, in WIPΔABD mutant B cells after BCR stimulation, we went on to analyze the influence of WIP binding to actin on CD19 diffusion on the resting B cell surface. For this, we performed single-particle tracking (SPT) of CD19 by incubating primary B cells with limited amounts of fluorescently labeled anti-CD19 Fab fragments, followed by analysis with total internal reflection fluorescence (TIRF) microscopy. Unlike CD19 particles on *Wipf1*^−/−^ B cells, CD19 particles on the surface of WIPΔABD mutant B cells were relatively immobile, with the median diffusion coefficient only slightly increasing from 0.009 μm^2^/s on WT B cells to 0.012 μm^2^/s on WIPΔABD mutant B cells (Figures 3A and 3B; Video S1). Two populations of CD19 particles on the surface of B cells could be distinguished based on a higher and lower mobility. This more detailed analysis revealed a shift of CD19 molecules to the population retaining higher mobility on the surface of WIPΔABD mutant B cells, with an increase from 36% on WT B cells to 55% on WIPΔABD mutant B cells (Figures 3C and 3D).

Video S1. Diffusion of CD19 Particles on the Surface of WT and WIPΔABD B Cells, Related to Figure 3 TIRF microscopy visualization of the diffusion of single particles of CD19 under nonstimulatory conditions on WT and WIPΔABD B cells. Images were collected every 50 ms and rebuilt at 20 frames/s. The 2D tracks of particles were color coded. Scale Bar = 2 μm.

We next analyzed CD19 and CD81 expression levels on the surface of WIPΔABD mutant and WT B cells. In line with F-actin levels (Figure 2B), CD19 expression was comparable between the two cell types (Figure 3E). However, the expression of the tetraspanin protein CD81 on the surface of WIPΔABD mutant was often reduced (Figure 3F). These results indicate that also on WIPΔABD mutant B cells, the reduced CD81 levels correlated with an increase in the more mobile population of CD19 molecules, likely leading to a misplacement of the co-receptor and resulting in impaired signaling.

### Binding of WIP to Actin Affects B Cell Chemotaxis

**Figure 4:**
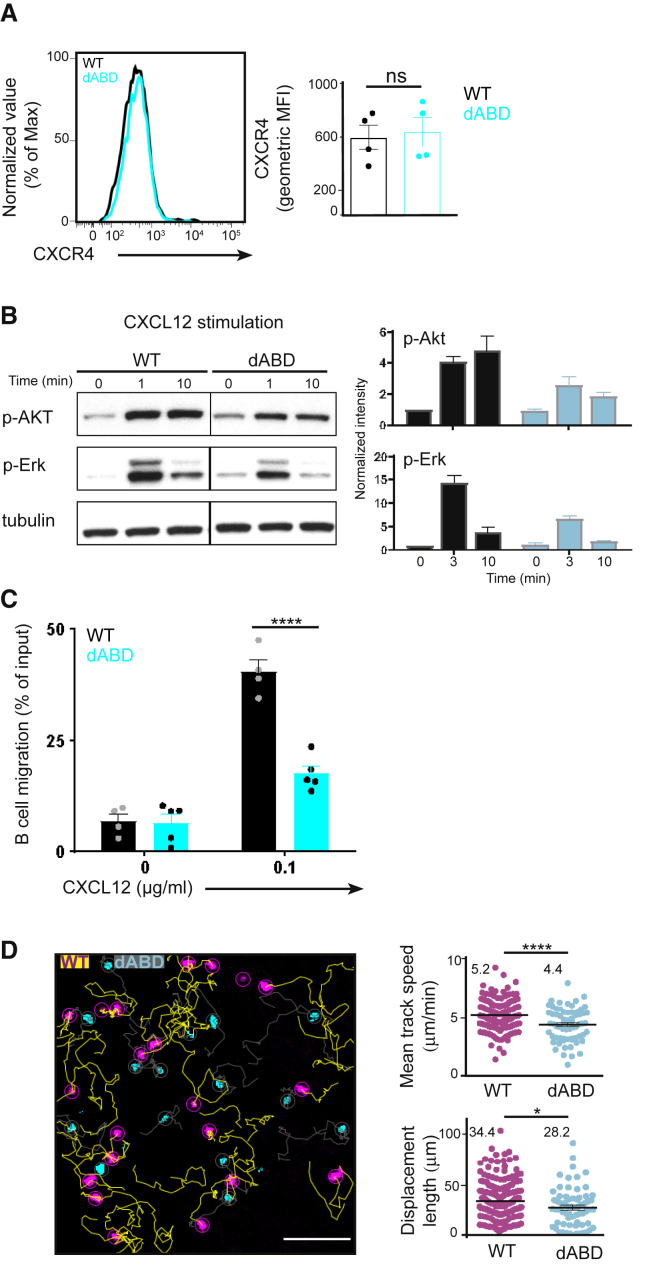
WIP Binding to Actin Regulates B Cell Chemotaxis (A) Flow cytometric analysis of CXCR4 expression on the surface of WT and WIPΔABD B cells. Quantification on the right shows the gMFI (mean ± SEM). (B) Immunoblot of splenic WT and WIPΔABD B cells treated with CXCL12 and probed with antibodies as indicated. Quantifications of intensity of proteins normalized by densitometry to tubulin and to the signal in unstimulated WT cells at t = 0. Data (mean ± SEM) are representative of at least three independent experiments. (C) Chemotactic response of purified WT and WIPΔABD B cells to CXCL12 using transwell plates. Graphs (± SEM) show percentages of migrating cells normalized to the starting input number of B cells set to 100%. Data (mean ± SEM) are pooled from four independent experiments. (D) Representative two-photon image of homeostatic movement 24 hr after adoptive transfer of labeled WT (magenta, yellow tails) and WIPΔABD (cyan, gray tails) B cells (scale bar, 300 μm). Graphs on the right indicate the quantification of the average speed over the span of the video and the mean displacement length. Data (mean ± SEM) are representative of two independent experiments. See also Video S2.

Cytoskeletal integrity is crucial for B cell migration and chemotaxis. Having observed a slightly hampered reconstitution capacity of WIPΔABD mutant BM in chimeric mice (Figures S1A and S1D), we went on to investigate signaling after CXCR4 stimulation. First, we determined that surface expression of CXCR4 was similar on WIPΔABD mutant and WT B cells (Figure 4A). Next, we stimulated WIPΔABD mutant and WT B cells with the CXCR4 ligand CXCL12 and analyzed the phosphorylation of Akt and Erk by immunoblot. WIPΔABD mutant B cells showed consistently diminished phosphorylation of Akt and Erk compared to WT B cells (Figure 4B).

We next evaluated the B cell chemotactic response to the chemokine CXCL12 *in vitro* using transwell plates. WIPΔABD mutant B cells displayed a significant reduction in the percentage of migratory B cells to CXCL12 compared to WT cells (Figure 4C). We went on to investigate the steady-state migration of naive B cells *in vivo*. For this, fluorescently labeled WT and WIPΔABD mutant B cells were adoptively transferred into WT recipients. After 24 hr, both cell types were found in explanted popliteal and inguinal lymph nodes using two-photon microscopy. Although WT B cells moved with an average speed of 5.2 ± 0.076 μm/min, WIPΔABD mutant B cells were significantly slower at 4.4 ± 0.173 μm/min (Figure 4D; Video S2). In addition, WIPΔABD mutant B cells showed about 20% less displacement during the acquisition period compared to WT cells, thus indicating a reduced steady-state migration of these cells.

Video S2. Migration of WT and WIPΔABD B Cells in an Explanted Popliteal Lymph Node, Related to Figure 4 Multiphoton microscopy time-lapse imaging of CTV-labeled WT (magenta) and CFSE-labeled WIPΔABD B (cyan) B cells injected in a WT recipient animal and migrating in an explanted popliteal lymph node. Migration of individual cells was tracked over time in part of a lymph node. Scale Bar = 20 μm.

Altogether, these results demonstrated that the absence of WIP binding to actin influences B cell migration and actin polarization, likely affecting B cell function.

### Defective Polarization and HS1 Accumulation in B Cells Lacking Binding of WIP to Actin

**Figure 5:**
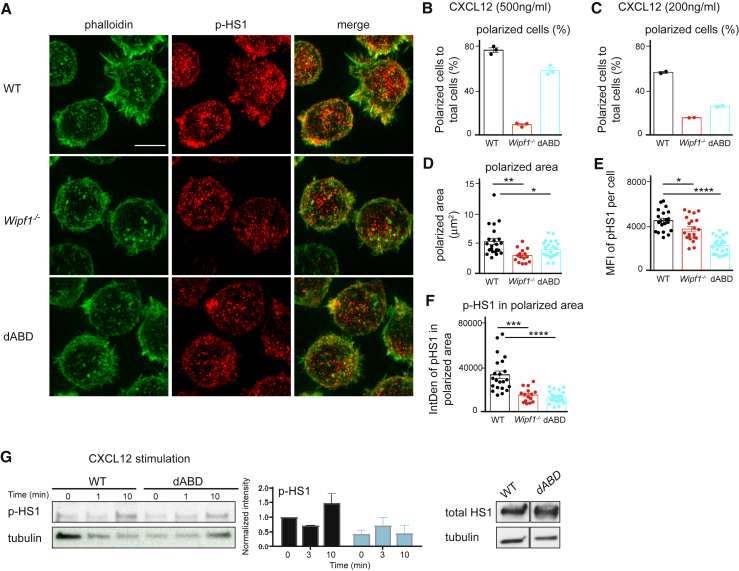
Defective Polarization and p-HS1 Accumulation in B Cells Lacking the ABD of WIP (A) Representative SIM images (maximum intensity projections) of phalloidin and p-HS1 staining in fixed WT, *Wipf1*^−/−^, and WIPΔABD B cells settled on coverslips coated with poly-L-lysin and treated with CXCL12 (500 ng/mL) for 3 min. Scale bar, 4 μm. (B and C) Quantifications show the percentage (mean ± SEM) of polarized cells (each dot represents an image with about 50 cells each) after stimulation with (B) 500 ng/mL or (C) 200 ng/mL CXCL12. (D–F) Quantification of the (D) polarized area, (E) MFI of p-HS1 per cell, and (F) fluorescence intensity of p-HS1 in the polarized area as analyzed by the ImageJ software. Each dot represents a cell. About 150 cells were analyzed per genotype. Data (mean ± SEM) are representative of at least two independent experiments. (G) Immunoblot of splenic WT and WIPΔABD B cells treated with CXCL12 and probed with antibodies against p-HS1, HS1, and tubulin. Data (mean ± SEM) are representative of two independent experiments. See also Figure S3.

Having observed a reduced chemotaxis and diminished PI3K signaling after CXCL12 stimulation *in vitro*, we next investigated actin polarization of WIPΔABD mutant B cells upon chemokine stimulation. For this, cells were spread on nonactivating coverslips, stimulated with CXCL12, fixed, stained for F-actin by phalloidin, and imaged using SIM. After 3 min of stimulation, WT B cells showed lamellipodia formation as polarized F-actin structures (Figure 5A; Figure S3A). In contrast, both *Wipf1*^−/−^ and WIPΔABD mutant B cells failed to polarize F-actin after CXCL12 stimulation, indicated as the percentage polarized cells to total cells (Figures 5A–5D). This defect in actin polarization depended on the strength of the stimulus, because stimulation with a higher dose of CXCL12 led to more polarized WIPΔABD mutant B cells, but not *Wipf1*^−/−^ B cells (compare Figure 5B [500 ng/mL] to Figure 5C [200 ng/mL]).

WIP has been shown to interact with cortactin, an actin binding protein that promotes actin polymerization by stabilizing branched actin filaments (Weaver et al., 2001), in a yeast-two hybrid screen (Kinley et al., 2003). We thus went on to investigate whether HS1, the hematopoietic homolog of cortactin, localizes to actin-rich structures after CXCR4 activation in B cells. SIM imaging of CXCL12-stimulated WT cells revealed an increase in HS1 phosphorylation, as well as accumulation of activated HS1 in areas of polarized actin, 3 min after activation, as indicated by the increase in MFI of p-HS1 in WT cells (Figures S3A and S3B). Concomitantly to the reduced ability of *Wipf1*^−/−^ and WIPΔABD mutant B cells to polarize F-actin after CXCL12 stimulation, both cell types showed reduced phosphorylation and polarization of HS1 (Figure 5A; Figure S3C), as detected by SIM imaging. MFI analysis of p-HS1 staining per cell confirmed a diminished overall phosphorylation of HS1 in *Wipf1*^−/−^ and WIPΔABD mutant B cells compared to WT cells 3 min after CXCL12 stimulation (Figure 5E; Figure S3D). Similarly, phosphorylation levels of HS1 were reduced after stimulation of WIPΔABD mutant B cells with CXCL12 or BCR, as detected by immunoblot (Figure 5G; Figure S3F). Furthermore, we detected more of the phosphorylated and thus activated form of HS1 polarized in actin-rich areas in CXCL12-treated WT B cells, but not in *Wipf1*^−/−^ and less in WIPΔABD mutant B cells, as indicated by the intensity of p-HS1 staining within the actin-rich area (Int Den, the product of area and mean gray value of p-HS1) (Figure 5F; Figure S3E). From these data, we conclude that the binding of WIP to actin is necessary for actin polarization after CXCL12 stimulation. Furthermore, phosphorylation and polarization of HS1 is reduced in WIPΔABD mutant B cells, likely indicating a crucial role for the binding of WIP to actin in regulating HS1 activation and hence B cell chemotaxis.

## Discussion

The results presented in this study elucidated a crucial role of the binding of WIP to actin for B cell function. Immunizing mixed BM chimeras revealed that in the absence of WIP binding to actin, B cells are less efficient at differentiating into GC B cells in a competitive environment. However, in a noncompetitive environment, GC responses are comparable to WT cells but are impaired in producing high-affinity antibodies. These results suggest a crucial role of WIP binding to actin during B cell differentiation and antibody production. B cells deficient for WASp mount a normal antibody response to T-dependent immunization (Recher et al., 2012, Snapper et al., 1998). This can be partly explained by compensatory effects of the ubiquitously expressed protein N-WASp (Westerberg et al., 2012), because mice lacking both WASp and N-WASp in B cells have impaired responses to T cell-dependent antigens (Volpi et al., 2016). Considering our previously published data, we now suggest that in *Wipf1*^−/−^ B cells, the lack of differentiation into GC cells and antibody production can be attributed to the strongly reduced PI3K signaling and resulting defects in survival and differentiation due to the absence of both WIP and WASp. In contrast, B cells lacking the ABD of WIP are less fit to enter the GC response in a competitive environment and demonstrate enhanced class-switched recombination but reduced antibody affinity maturation. Findings demonstrate that clonal expansion of high-affinity B cells in GCs depends on the degradation of IRF4 in light zone B cells (Li et al., 2018). We found that WIPΔABD B cells preferentially differentiate into antigen-specific, IRF4-expressing, IgG1-high B cells *in vivo*. We speculate that these cells could be plasma cells that, because of enhanced IRF4 levels, prematurely exit the GC reaction, having undergone less affinity maturation, and secrete increased levels of IgG1 in the serum. How the binding of WIP to actin regulates IRF4 expression levels remains to be investigated.

One of the reasons for the observed altered antibody response in WIPΔABD mice could be the reduced PI3K signaling we detected after BCR stimulation. In *Wipf1*^−/−^ B cells, we have previously demonstrated a severe defect in PI3K signaling after triggering the BCR but also other receptors (Keppler et al., 2015). We show here that WIPΔABD B cells demonstrate less severe defects in signaling after BCR or CXCR4 stimulation compared to *Wipf1*^−/−^ B cells. Phosphorylation of CD19 is reduced in both *Wipf1^−/−^* and WIPΔABD B cells. These results indicate that despite less severe signaling defects in WIPΔABD B cells, CD19 phosphorylation depends on WIP binding to actin.

We and others have shown that the small Rho guanosine triphosphatases (GTPases) Cdc42, a known regulator of actin remodeling and interaction partner of WASp and N-WASp, and to a lesser extent TC10 control GC formation and antibody production in B cells (Burbage et al., 2015, Burbage et al., 2017, Gerasimcik et al., 2015). Furthermore, the lack of the adaptor protein and known interaction partner of WIP, WASP, and Cdc42 Intersectin2 demonstrated reduced antigen affinity maturation after vaccination (Burbage et al., 2018). Together with the findings presented here, these results point at a central function of actin regulators in influencing humoral immune responses. A comparison of the B cell functions dependent on WIP or the binding of WIP to actin is shown in Table S1.

In the absence of WIP, and hence WASp, we have demonstrated that antigen-induced B cell synapses showed a loss of actin foci (Keppler et al., 2015). Similar F-actin foci were described as being a WASp-generated fraction of the total synaptic F-actin in T cells (Kumari et al., 2015). The results presented here revealed that although overall F-actin levels in resting WT and WIPΔABD mutant B cells were similar, WIPΔABD mutant B cells were less able to form F-actin foci after BCR stimulation or polarization of F-actin after triggering the chemokine receptor CXCR4. In line with this, T cells from WIPΔABD mutant mice were impaired in their capacity to spread over anti-CD3-coated coverslips (Massaad et al., 2014). Altogether, these findings indicate that the binding of WIP to actin is necessary for actin polymerization after BCR and chemokine receptor stimulation, especially in situations that require active actin reorganization of the cells.

We previously demonstrated that in the absence of WIP, the actin cytoskeleton, as well as the CD81 tetraspanin network, was disturbed, leading to increased diffusion of CD19 on resting B cells and resulting in impaired PI3K signaling (Keppler et al., 2015). Employing SPT to study CD19 diffusion on the resting WIPΔABD mutant B cell surface, we here surprisingly found a fraction of CD19 molecules to be highly mobile, although the overall CD19 diffusion was similar to WT B cells. This increased CD19 diffusion correlated with reduced expression of the tetraspanin molecule CD81 on WIPΔABD mutant B cells. These results point at a function of WIP binding to actin in stabilizing the tetraspanin molecule CD81, thereby regulating CD19 diffusion. How these subtle changes in the actin cytoskeleton through the binding of WIP to actin influence the plasma membrane expression of CD81 remains to be investigated. A higher mobility of a fraction of CD19 molecules, as well as reduced actin polymerization, might be the underlying mechanism for reduced PI3K signaling of WIPΔABD mutant B cells after triggering the BCR or CXCR4. The polymerization of actin after the initial receptor-mediated depolymerization occurs at BCR microclusters and is needed to stabilize BCR molecules close CD19 molecules (Kläsener et al., 2014). The formation, integrity, and proximal signaling events of these microclusters depend on the actin cytoskeleton (Treanor et al., 2011). Altered receptor dynamics, such as seen in the absence of WIP or WIP binding to actin, might hamper receptor crosstalk and limit CD19 in its function in PI3K signaling.

Analyzing the reconstitution capacity of WIPΔABD mutant BM, we found reduced reconstitution of B cells in the BM of recipient mice, similar to what we have described with BM from WIP-deficient mice. Furthermore, we demonstrated here that WIPΔABD mutant B cells showed reduced homeostatic displacement in peripheral lymphoid organs *in vivo* and reduced chemotaxis toward CXCL12 *in vitro*. Concomitantly, PI3K signaling was impaired in WIPΔABD mutant B cells after triggering CXCR4. Similarly, T cells lacking the ABD of WIP displayed decreased cellular F-actin content, impaired chemotaxis, and defective homing to lymph nodes despite having normal WASp expression (Massaad et al., 2014). Further to our finding that an immobile co-receptor CD19 is necessary for PI3K signaling and chemotaxis (Keppler et al., 2015), it has been proposed that CXCR4 signaling in B cells requires the expression of the BCR and that triggering CD19 can overcome this need (Becker et al., 2017). We here show that even small alterations in the mobile fraction of CD19 triggered by the absence of WIP binding to actin can lead to reduced PI3K signaling and chemotaxis of B cells, demonstrating once more the crucial role of CD19 and the actin cytoskeleton in regulating B cell chemotactic responses.

Branched actin networks in lymphocytes are not only regulated by the WASp family of actin regulators but also by HS1. HS1 stabilizes the ARP2/3 complex against debranching (Weaver et al., 2001) and interacts with WASp and WIP in dendritic cells to ensure chemotaxis (Dehring et al., 2011, Kinley et al., 2003). Furthermore, HS1 is essential for T cell receptor (TCR) synapse formation (Gomez et al., 2006). HS1 is phosphorylated after BCR stimulation and is required for B cell responses to antigens (Taniuchi et al., 1995). However, the role of HS1 in B cell chemotaxis is unknown so far. Here, by using SIM, we demonstrated that HS1 is polarized to F-actin-rich structures after CXCL12 stimulation. In the absence of WIP or WIP binding to actin, B cells failed to polarize F-actin or HS1 in response to CXCL12. We thus hypothesize that to migrate toward a chemokine gradient, B cells require WIP binding to actin for stabilization of branched actin filaments by HS1. This might be a direct effect of WIP recruiting active p-HS1 or leading to the activation of HS1 or, alternatively, an indirect activation through actin.

Besides stabilizing WASp and binding to F-actin filaments, WIP associates with the adaptor molecules Nck, Grb2, and DOCK8, thus linking the actin network to signaling cascades (Antón et al., 1998, Barda-Saad et al., 2005, Donnelly et al., 2013, Janssen et al., 2016, Moreau et al., 2000). We here extend this view of WIP as an organizer of signaling platforms by proposing a model in which WIP is essential for maintaining actin cytoskeleton integrity and promoting migration: it stabilizes WASp and stabilizes newly synthesized actin filaments by recruiting or activating HS1. A better understanding of these processes will help modulate actin cytoskeletal functions with the aim of preventing or improving disease.

## Experimental Procedures

### Animal Breeding and Generation

*Wipf1*^−/−^ mice (Antón et al., 2002) and WIPΔABD mice (Massaad et al., 2014) were gifts from Raif Geha (Boston’s Children Hospital, Boston, USA); Igh-J^tm1Dhu^ mice (BALB/c JHT) (Chen et al., 1993) were purchased from Taconic Bioscience; and CBy.SJL(B6)-Ptprc^a^/J mice (BALB/c CD45.1) were purchased from the Jackson Laboratory. BALB/c WT mice were obtained from the breeding facility at The Crick Institute. Age- and sex-matched mice of 8–12 weeks were used for experiments. All experiments were approved by the Animal Ethics Committee of the Francis Crick Institute and the United Kingdom Home Office.

### BM Chimeras

BM was harvested from femora and tibiae of donor mice. BALB/c JHT or BALB/c CD45.1 mice (recipients) were irradiated with 2 × 6.6 Gy and injected intravenously the day after with 2 × 10^6^ BM cells. Repopulation was determined and chimeras were used 8–10 weeks after injection.

### Immunization and ELISA

Mice were injected intraperitoneally (i.p.) with 50 μg of NP33-KLH (Biosearch Technology) in 4 mg of alum (Thermo Scientific). Blood samples were taken from the lateral tail vein on days 0, 4, 7, 14, and 21 after immunization. NP-specific antibody titers were detected by ELISA, using NP_29_-BSA or NP_4_-BSA for capture and biotinylated anti-mouse IgM (Southern Biotech), IgG (Southern Biotech), IgG1 (Southern Biotech), and IgG3 (BD Biosciences) for detection. Titers were determined from the dilution curve in the linear range of absorbance. ELISA plates were developed with alkaline-phosphatase streptavidin (Sigma) and phosphorylated nitrophenyl phosphate (Sigma). Absorbance at 405 nm was determined with a SPECTRAmax190 plate reader (Molecular Devices).

### Flow Cytometry

Single-cell suspensions from blood, spleen, lymph nodes, or BM were treated with lysis buffer to remove red blood cells before staining in fluorescence-activated cell sorting (FACS) buffer (PBS, 2% FCS). After blocking Fc receptors using anti-CD16/32 antibodies, cells were stained with the appropriate combination of the following antibodies (indicating antigen and clone): B220 (RA3-6B2), B and T cell activation antigen (GL7), CD95 (Jo2), CD19 (1D3), IgM (1B4B1 or II/41), IgD (11-26), IRF4 (3E4), CD45.1 (A20), CD45.2 (104), CXCR4 (2B11), CD21 (7G6), CD23 (B3B4), CD81 (EAT-2), and Alexa Fluor 488 phalloidin (Molecular Probes). Cells were acquired on LSRFortessa (BD Biosciences) and analyzed with FlowJo (Tree Star).

### Cell Isolation, Labeling, and Culture

Splenic naive B cells were purified using negative B cell isolation kits yielding enriched populations of about 95%–98% (Miltenyi). For transwell chemotaxis assays, transwell plates containing a 5 μm pore size membrane (Corning Costar) were used. 10^5^ purified splenic B cells were loaded onto the transwell filter in migration medium (RPMI, 0.5% BSA, and 10 mM HEPES). The chemokine CXCL12 was added at the indicated concentrations to the lower chamber. After 4 hr at 37°C, the transmigrating population was collected and counted by flow cytometry. Migration frequency was estimated as [(B cell number at the lower chamber / initial B cell input in the upper chamber) × 100] in each condition.

### Immunoblotting

After equilibration at 37°C for 10 min in chamber buffer (PBS, 0.5% FCS, 1 g/L D-glucose, 2 mM MgCl_2_, and 0.5 mM CaCl_2_), purified B cells were stimulated for indicated times with 3 μg/mL of anti-κ (HB-58, ATCC), 2.5 ng/mL of CXCL12 (R&D Systems), or anti-κ-coated microspheres (streptavidin-coated microspheres, 0.1 μm in diameter (Bangs Laboratories); incubated with a saturating amount of biotinylated anti-κ-chain antibody 1 hr at room temperature; and washed extensively before use.

Stimulated cells were lysed in lysis buffer (20 mM Tris-HCL [pH 8], 150 mM NaCl, 5 mM EDTA, protease inhibitor cocktail (Roche), 10 mM NaF, 1 mM Na_3_VO_4_, and 1% NP40) for 30 min on ice. After removal of cell debris by centrifugation, proteins of cell lysates were separated on 4%–20% or 12% SDS-PAGE gels (Mini-PROTEAN TGX, Bio-Rad) and transferred onto Trans-Blot Turbo polyvinylidene fluoride (PVDF) membrane (Bio-Rad). Membranes were blocked for 1 hr in 5% milk buffer and then probed with the following antibodies: p-Erk (Thr202/Tyr204) no. 9101, p-Akt (S473) no. 4058, p-CD19 (Tyr531) no. 3571, p-HS1 (Tyr397) no. 8714 (all from Cell Signaling Technology), p-WIP (S488) (BD Phosflow), and α-tubulin (Sigma no. 9026) using the secondary horseradish peroxidase (HRP)-conjugated anti-rabbit or anti-mouse antibodies (Jackson Laboratory). Blot densitometry analysis was performed using ImageJ software.

### Sample Preparation for SPT

Fab fragments of purified monoclonal antibodies to murine CD19 (1D3) were digested as previously described (Depoil et al., 2008). The glass coverslips for TIRF microscopy were cleaned in chromosulfuric acid (VWR), coated with 1 μg/mL of anti-major histocompatibility complex class II (anti-MHC class II) (M5/114, ATCC TIB120) for nonstimulatory coating for 1.5 hr, and washed with PBS.

TIRF images were acquired with an electron multiplying charge-coupled device (EMCCD) camera (iXon3 897, Andor) coupled to a TIRF microscopy system (Cell R, Olympus) with 488-, 561-, and 640-nm lasers (Olympus) and a motorized filter wheel (Olympus). Single-molecule imaging was performed as previously described with 20 frames/s for 15 s using a 150× numerical aperture (NA) 1.45 objective (Olympus). Images were recorded with Cell R software (Olympus) and analyzed with ImageJ software (NIH). SPT data were analyzed in MATLAB (MathWorks, Natick, MA), and population analysis was performed as previously described (Mattila et al., 2013, Treanor et al., 2010).

### SIM

SIM was performed on an Elyra PS.1 microscope (Zeiss) using 488- and 640-nm laser excitation and a 63×/1.40 Plan Apochromat oil-immersion objective (Zeiss). Imaging was started 20 min after placing the dish on the microscope to prevent artifacts due to sample drift. Two-color alignment was performed after each experiment day using a multicolor bead sample (Zeiss) and the channel alignment function in the Zen software (Zeiss). Images were reconstructed in the Zen software using a theoretical point-spread function and the auto-noise filter setting.

For spreading analysis, primary purified B cells were settled on coverslips coated with anti-κ-chain antibody at 37°C. After 10 min, cells were fixed with 2% paraformaldehyde, permeabilized with PBS 0.3% Triton X-100, and stained with Alexa Fluor 488 phalloidin (Molecular Probes). F-actin foci were calculated using the Analyze Particles plugin of ImageJ.

For chemotactic F-actin polarization assays, primary purified B cells were settled on coverslips coated with poly-L-lysin at 37°C. After 10 min, CXCL12 was added to the cells at the indicated concentrations. After 3 min of stimulation, cells were fixed with 2% paraformaldehyde, permeabilized with PBS 0.3% Triton X-100, and stained with Alexa Fluor 488 phalloidin (Molecular Probes) and p-HS1 (Cell Signaling Technology), followed by secondary antibody staining (anti-rabbit Alexa 555, Molecular Probes). The total cell number in a field of view was counted, as well as cells with lamellipodia (actin polarized), and the percentage of polarized cells to total cells was calculated. The area of actin polarization was circled manually in ImageJ, and area and the fluorescence intensity of p-HS1 in this area were blotted as given by ImageJ.

### Two-Photon Microscopy

Purified B cells were labeled in PBS with 1 μM cell trace violet (CTV) (Invitrogen) or 1 μM carboxyfluorescein succinimidyl ester (CFSE) (Invitrogen) for 10 min at 37°C and washed, and a mixture of differently labeled 2 × 10^6^ WT and WIPΔABD cells were adoptively transferred into age- and sex-matched recipients by tail vein injection. Colors were exchanged between cell types in each experiment. 24 hr after transfer, explanted popliteal and inguinal lymph nodes were prepared as described (Carrasco and Batista, 2007) and imaged with an upright multiphoton microscope (Olympus), a 25× NA 1.05 water immersion objective, and a pulsed Ti:sapphire laser (Spectra Physics MaiTai HP DeepSee) tuned to 800 nm. Emission wavelengths were detected through band-pass filters of 420–500 nm (CTV) and 515–560 nm (CFSE). Fluorescence bleed-through into the channel with the longer wavelength was removed by subtracting the channel with the shorter wavelength. Multidimensional videos were analyzed with Imaris software (Bitplane).

### Statistics

p values were determined by Student’s two-tailed t test for independent samples on all experimental datasets with Microsoft Excel or Prism 6. All data are shown as means ± SEM. Significant differences are indicated with ^∗^p < 0.05, ^∗∗^p < 0.01, ^∗∗∗^p < 0.001, and ^∗∗∗∗^p < 0.0001 or NS, not significant.

## Supplemental Information

Document S1. Figures S1–S3 and Table S1

Document S2. Article plus Supplemental Information

## References

[bib1] Antón I.M., Lu W., Mayer B.J., Ramesh N., Geha R.S. (1998). The Wiskott-Aldrich syndrome protein-interacting protein (WIP) binds to the adaptor protein Nck. J. Biol. Chem..

[bib2] Antón I.M., de la Fuente M.A., Sims T.N., Freeman S., Ramesh N., Hartwig J.H., Dustin M.L., Geha R.S. (2002). WIP deficiency reveals a differential role for WIP and the actin cytoskeleton in T and B cell activation. Immunity.

[bib3] Antón I.M., Saville S.P., Byrne M.J., Curcio C., Ramesh N., Hartwig J.H., Geha R.S. (2003). WIP participates in actin reorganization and ruffle formation induced by PDGF. J. Cell Sci..

[bib4] Barda-Saad M., Braiman A., Titerence R., Bunnell S.C., Barr V.A., Samelson L.E. (2005). Dynamic molecular interactions linking the T cell antigen receptor to the actin cytoskeleton. Nat. Immunol..

[bib5] Becker M., Hobeika E., Jumaa H., Reth M., Maity P.C. (2017). CXCR4 signaling and function require the expression of the IgD-class B-cell antigen receptor. Proc. Natl. Acad. Sci. USA.

[bib6] Burbage M., Keppler S.J., Gasparrini F., Martinez-Martin N., Gaya M., Feest C., Domart M.C., Brakebusch C., Collinson L., Bruckbauer A., Batista F.D. (2015). Cdc42 is a key regulator of B cell differentiation and is required for antiviral humoral immunity. J. Exp. Med..

[bib7] Burbage M., Keppler S.J., Montaner B., Mattila P.K., Batista F.D. (2017). The csmall Rho GTPase TC10 modulates B cell immune responses. J. Immunol..

[bib8] Burbage M., Gasparrini F., Aggarwal S., Gaya M., Arnold J., Nair U., Way M., Bruckbauer A., Batista F.D. (2018). Tuning of *in vivo* cognate B-T cell interactions by Intersectin 2 is required for effective anti-viral B cell immunity. eLife.

[bib9] Carrasco Y.R., Batista F.D. (2007). B cells acquire particulate antigen in a macrophage-rich area at the boundary between the follicle and the subcapsular sinus of the lymph node. Immunity.

[bib10] Chen J., Trounstine M., Alt F.W., Young F., Kurahara C., Loring J.F., Huszar D. (1993). Immunoglobulin gene rearrangement in B cell deficient mice generated by targeted deletion of the JH locus. Int. Immunol..

[bib11] Dehring D.A., Clarke F., Ricart B.G., Huang Y., Gomez T.S., Williamson E.K., Hammer D.A., Billadeau D.D., Argon Y., Burkhardt J.K. (2011). Hematopoietic lineage cell-specific protein 1 functions in concert with the Wiskott-Aldrich syndrome protein to promote podosome array organization and chemotaxis in dendritic cells. J. Immunol..

[bib12] Depoil D., Fleire S., Treanor B.L., Weber M., Harwood N.E., Marchbank K.L., Tybulewicz V.L., Batista F.D. (2008). CD19 is essential for B cell activation by promoting B cell receptor-antigen microcluster formation in response to membrane-bound ligand. Nat. Immunol.

[bib13] Donnelly S.K., Weisswange I., Zettl M., Way M. (2013). WIP provides an essential link between Nck and N-WASP during Arp2/3-dependent actin polymerization. Curr. Biol..

[bib14] Fried S., Matalon O., Noy E., Barda-Saad M. (2014). WIP: more than a WASp-interacting protein. J. Leukoc. Biol..

[bib15] Gallego M.D., de la Fuente M.A., Anton I.M., Snapper S., Fuhlbrigge R., Geha R.S. (2006). WIP and WASP play complementary roles in T cell homing and chemotaxis to SDF-1alpha. Int. Immunol..

[bib16] Gasparrini F., Feest C., Bruckbauer A., Mattila P.K., Müller J., Nitschke L., Bray D., Batista F.D. (2016). Nanoscale organization and dynamics of the siglec CD22 cooperate with the cytoskeleton in restraining BCR signalling. EMBO J..

[bib17] Gerasimcik N., Dahlberg C.I., Baptista M.A., Massaad M.J., Geha R.S., Westerberg L.S., Severinson E. (2015). The Rho GTPase Cdc42 is essential for the activation and function of mature B cells. J. Immunol..

[bib18] Gomez T.S., McCarney S.D., Carrizosa E., Labno C.M., Comiskey E.O., Nolz J.C., Zhu P., Freedman B.D., Clark M.R., Rawlings D.J. (2006). HS1 functions as an essential actin-regulatory adaptor protein at the immune synapse. Immunity.

[bib19] Hao S., August A. (2005). Actin depolymerization transduces the strength of B-cell receptor stimulation. Mol. Biol. Cell.

[bib20] Janssen E., Tohme M., Hedayat M., Leick M., Kumari S., Ramesh N., Massaad M.J., Ullas S., Azcutia V., Goodnow C.C. (2016). A DOCK8-WIP-WASp complex links T cell receptors to the actin cytoskeleton. J. Clin. Invest..

[bib21] Keppler S.J., Gasparrini F., Burbage M., Aggarwal S., Frederico B., Geha R.S., Way M., Bruckbauer A., Batista F.D. (2015). Wiskott-Aldrich syndrome interacting protein deficiency uncovers the role of the co-receptor CD19 as a generic hub for PI3 kinase signaling in B cells. Immunity.

[bib22] Kinley A.W., Weed S.A., Weaver A.M., Karginov A.V., Bissonette E., Cooper J.A., Parsons J.T. (2003). Cortactin interacts with WIP in regulating Arp2/3 activation and membrane protrusion. Curr. Biol..

[bib23] Kläsener K., Maity P.C., Hobeika E., Yang J., Reth M. (2014). B cell activation involves nanoscale receptor reorganizations and inside-out signaling by Syk. eLife.

[bib24] Kumari S., Depoil D., Martinelli R., Judokusumo E., Carmona G., Gertler F.B., Kam L.C., Carman C.V., Burkhardt J.K., Irvine D.J., Dustin M.L. (2015). Actin foci facilitate activation of the phospholipase C-γ in primary T lymphocytes via the WASP pathway. eLife.

[bib25] Lanzi G., Moratto D., Vairo D., Masneri S., Delmonte O., Paganini T., Parolini S., Tabellini G., Mazza C., Savoldi G. (2012). A novel primary human immunodeficiency due to deficiency in the WASP-interacting protein WIP. J. Exp. Med.

[bib26] Li X., Gadzinsky A., Gong L., Tong H., Calderon V., Li Y., Kitamura D., Klein U., Langdon W.Y., Hou F. (2018). Cbl ubiquitin ligases control B cell exit from the germinal-center reaction. Immunity.

[bib27] Martinez-Quiles N., Rohatgi R., Antón I.M., Medina M., Saville S.P., Miki H., Yamaguchi H., Takenawa T., Hartwig J.H., Geha R.S., Ramesh N. (2001). WIP regulates N-WASP-mediated actin polymerization and filopodium formation. Nat. Cell Biol..

[bib28] Massaad M.J., Oyoshi M.K., Kane J., Koduru S., Alcaide P., Nakamura F., Ramesh N., Luscinskas F.W., Hartwig J., Geha R.S. (2014). Binding of WIP to actin is essential for T cell actin cytoskeleton integrity and tissue homing. Mol. Cell. Biol..

[bib29] Mattila P.K., Feest C., Depoil D., Treanor B., Montaner B., Otipoby K.L., Carter R., Justement L.B., Bruckbauer A., Batista F.D. (2013). The actin and tetraspanin networks organize receptor nanoclusters to regulate B cell receptor-mediated signaling. Immunity.

[bib30] Mattila P.K., Batista F.D., Treanor B. (2016). Dynamics of the actin cytoskeleton mediates receptor cross talk: An emerging concept in tuning receptor signaling. J. Cell Biol..

[bib31] Moreau V., Frischknecht F., Reckmann I., Vincentelli R., Rabut G., Stewart D., Way M. (2000). A complex of N-WASP and WIP integrates signalling cascades that lead to actin polymerization. Nat. Cell Biol..

[bib32] Pfajfer L., Seidel M.G., Houmadi R., Rey-Barroso J., Hirschmugl T., Salzer E., Antón I.M., Urban C., Schwinger W., Boztug K., Dupré L. (2017). WIP deficiency severely affects human lymphocyte architecture during migration and synapse assembly. Blood.

[bib33] Ramesh N., Antón I.M., Hartwig J.H., Geha R.S. (1997). WIP, a protein associated with Wiskott-Aldrich syndrome protein, induces actin polymerization and redistribution in lymphoid cells. Proc. Natl. Acad. Sci. USA.

[bib34] Recher M., Burns S.O., de la Fuente M.A., Volpi S., Dahlberg C., Walter J.E., Moffitt K., Mathew D., Honke N., Lang P.A. (2012). B cell-intrinsic deficiency of the Wiskott-Aldrich syndrome protein (WASp) causes severe abnormalities of the peripheral B-cell compartment in mice. Blood.

[bib35] Snapper S.B., Rosen F.S., Mizoguchi E., Cohen P., Khan W., Liu C.H., Hagemann T.L., Kwan S.P., Ferrini R., Davidson L. (1998). Wiskott-Aldrich syndrome protein-deficient mice reveal a role for WASP in T but not B cell activation. Immunity.

[bib36] Song W., Liu C., Upadhyaya A. (2014). The pivotal position of the actin cytoskeleton in the initiation and regulation of B cell receptor activation. Biochim. Biophys. Acta.

[bib37] Stewart D.M., Tian L., Nelson D.L. (1999). Mutations that cause the Wiskott-Aldrich syndrome impair the interaction of Wiskott-Aldrich syndrome protein (WASP) with WASP interacting protein. J. Immunol..

[bib38] Taniuchi I., Kitamura D., Maekawa Y., Fukuda T., Kishi H., Watanabe T. (1995). Antigen-receptor induced clonal expansion and deletion of lymphocytes are impaired in mice lacking HS1 protein, a substrate of the antigen-receptor-coupled tyrosine kinases. EMBO J..

[bib39] Thrasher A.J. (2009). New insights into the biology of Wiskott-Aldrich syndrome (WAS). Hematology Am. Soc. Hematol. Educ. Program.

[bib40] Tolar P. (2017). Cytoskeletal control of B cell responses to antigens. Nat. Rev. Immunol..

[bib41] Treanor B., Depoil D., Gonzalez-Granja A., Barral P., Weber M., Dushek O., Bruckbauer A., Batista F.D. (2010). The membrane skeleton controls diffusion dynamics and signaling through the B cell receptor. Immunity.

[bib42] Treanor B., Depoil D., Bruckbauer A., Batista F.D. (2011). Dynamic cortical actin remodeling by ERM proteins controls BCR microcluster organization and integrity. J. Exp. Med.

[bib43] Van Troys M., Dewitte D., Goethals M., Carlier M.F., Vandekerckhove J., Ampe C. (1996). The actin binding site of thymosin beta 4 mapped by mutational analysis. EMBO J..

[bib44] Volpi S., Santori E., Abernethy K., Mizui M., Dahlberg C.I., Recher M., Capuder K., Csizmadia E., Ryan D., Mathew D. (2016). N-WASP is required for B-cell-mediated autoimmunity in Wiskott-Aldrich syndrome. Blood.

[bib45] Weaver A.M., Karginov A.V., Kinley A.W., Weed S.A., Li Y., Parsons J.T., Cooper J.A. (2001). Cortactin promotes and stabilizes Arp2/3-induced actin filament network formation. Curr. Biol..

[bib46] Westerberg L.S., Dahlberg C., Baptista M., Moran C.J., Detre C., Keszei M., Eston M.A., Alt F.W., Terhorst C., Notarangelo L.D., Snapper S.B. (2012). Wiskott-Aldrich syndrome protein (WASP) and N-WASP are critical for peripheral B-cell development and function. Blood.

