# Supplementary material for: The Lack of WIP Binding to Actin Results in Impaired B Cell Migration and Altered Humoral Immune Responses
Source: Cell Rep. 2018 Jul 17;24(3):619–29. doi: 10.1016/j.celrep.2018.06.051 (PMC6077251; doi:10.1016/j.celrep.2018.06.051)
Supplement: Document S1. Figures S1–S3 and Table S1 [file mmc1.pdf]

Cell Reports, Volume *24*

Supplemental Information

The Lack of WIP Binding to Actin Results in Impaired B Cell Migration and Altered Humoral Immune Responses

Selina Jessica Keppler, Marianne Burbage, Francesca Gasparrini, Lara Hartjes, Shweta Aggarwal, Michel J. Massaad, Raif S. Geha, Andreas Bruckbauer, and Facundo D. Batista

Title

The lack of WIP binding to actin results in impaired B cell migration and altered humoral immune responses

Authors

Selina Jessica Keppler, Marianne Burbage, Francesca Gasparrini, Lara Hartjes, Shweta Aggarwal, Michel J. Massaad, Raif S. Geha, Andreas Bruckbauer, Facundo D. Batista

Inventory of Supplemental Information

Main Text Figure 1 is supported by Supplemental Figure S1.
Main Text Figure 2 is supported by Supplemental Figure S2.
Main Text Figure 3 is supported by Supplemental Movie S1.
Main Text Figure 4 is supported by Supplemental Movie S2.
Main Text Figure 5 is supported by Supplemental Figure S3.

Main text Figures 1 – 5 are supported by Supplemental Table S1.

Supplemental Figure Legends:

Movie S1 – Related to Figure 3
TIRF microscopy visualization of the diffusion of single particles of CD19 under nonstimulatory conditions on WT and WIPΔABD B cells. Images were collected every 50 ms and rebuilt at 20 frames/s. The 2D tracks of particles were color coded. Scale Bar = 2 µm.

Movie S2 – Related to Figure 4
Multiphoton microscopy time-lapse imaging of CTV-labeled WT (magenta) and CFSE-labeled WIPΔABD B (cyan) B cells injected in a WT recipient animal and migrating in an explanted popliteal lymph node. Migration of individual cells was tracked over time in part of a lymph node. Scale Bar = 20 µm.

Figure S1 - related to Figure 1:

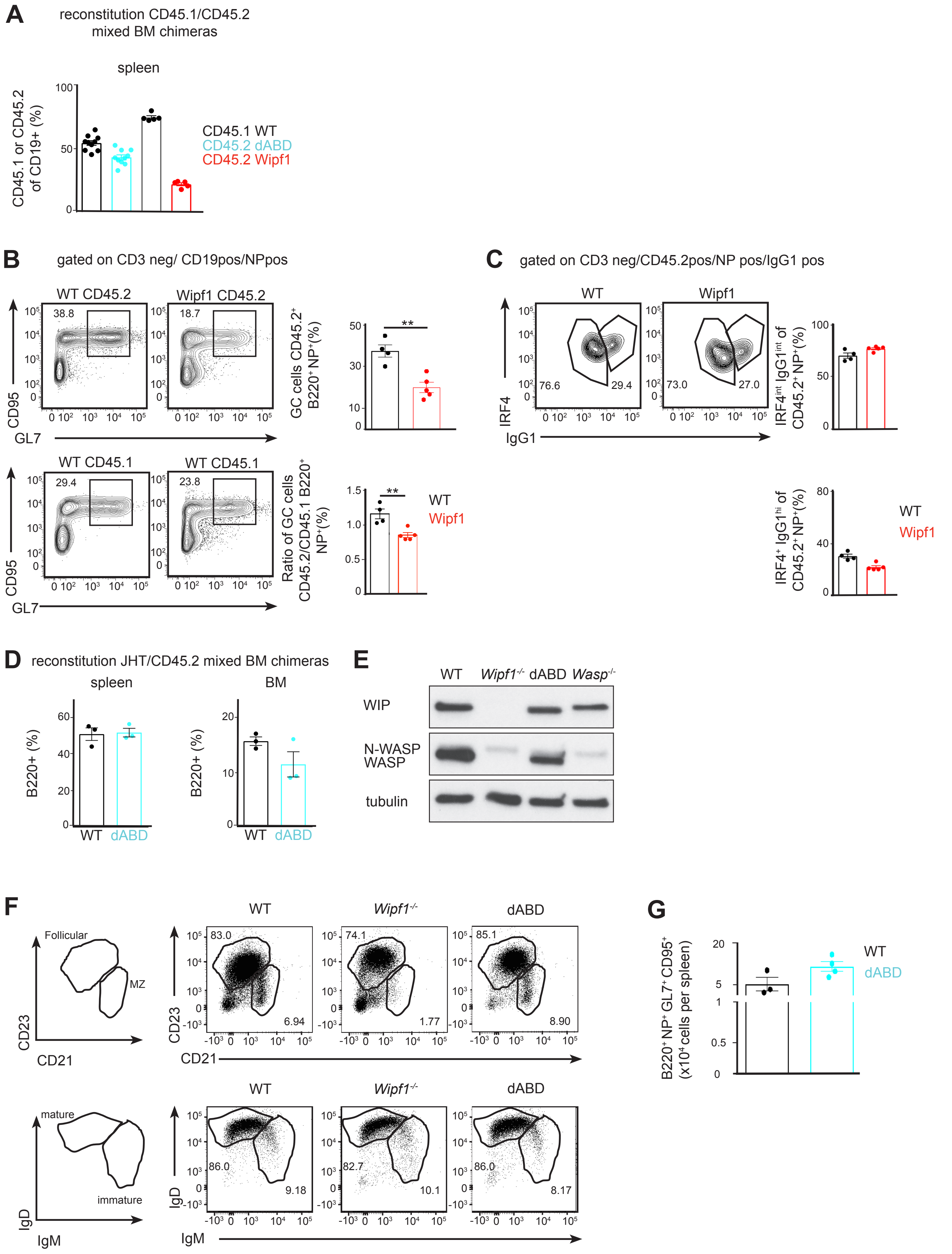

Figure S1 – related to Figure 1:

(A) Lethally irradiated CD45.1 recipients were reconstituted for 10 weeks with mixtures of 50% WT CD45.1$^+$ BM and 50% WT, *Wipf1$^{-/-}$* or WIPΔABD CD45.2$^+$ BM. Flow cytometric analysis of WT cells (CD19+CD45.1+, black) and WIPΔABD cells (B220+CD45.2+, cyan) as well as WT cells (CD19+CD45.1+, black) and WIP KO cells (B220+CD45.2+, red) in the spleen of mixed BM chimeras. Data are representative of 2 independent experiments with at least 4 mice per group.

(B+C) Mixed BM chimeras generated as in (A) were immunized with NP-KLH in alum. Spleens of immunized animals were analyzed by flow cytometry at day 13. For comparison, the same WT data as in Fig. 1A are shown. Data are representative of 2 independent experiments with at least 4 mice per group.

(B) CD45.1$^+$ and CD45.2$^+$ NP-specific GC B cells (B220$^+$, NP$^+$, GL7$^+$, CD95$^+$) are shown. Graphs on the right indicate percentages of CD45.2$^+$ GC cells and the CD45.2/CD45.1 ratio of GC B cells.

(C) CD45.2$^+$, NP-specific and IgG1$^+$ cells expressing the transcription factor IRF4 are shown. Graphs on the right indicate percentages of IRF4^{int}IgG1^{int} and IRF4$^+$IgG1^{hi} cells.

(D) Reconstitution analysis of the percentages of B220+ cells in JHT-WT or JHT- WIPΔABD mixed bone marrow chimeric mice in spleen and BM 10 weeks after irradiation.

(E) Representative Immunoblot of WIP, N-WASp and WASp (the same antibody recognizes both, N-WASp and WASp) and tubulin in lysates from purified splenic B cells from WT, *Wipf1$^{-/-}$*, WIPΔABD and *Wasp$^{-/-}$* mice.

(F) Spleens from WT, *Wipf1$^{-/-}$* and WIPΔABD mice were analyzed by flow cytometry. Follicular B cells (CD23^{hi}CD21^{lo}) and marginal zone B cells (MZ) (CD23^{lo}CD21^{hi}) are shown. Mature (IgM^{lo}IgD^{hi}) and immature (IgM^{hi}IgD^{lo}) B cell subsets were analyzed.

(G) JHT-WT, JHT-WIP KO or JHT-WIPΔABD mixed BM chimeric mice were immunized with NP-KLH in alum. Analysis of splenic NP-specific GC cells (B220$^+$, NP$^+$, GL7$^+$, CD95$^+$) at day 13 post-immunization by flow-cytometry. Quantifications show numbers of GC cells per spleen.

Figure S2 - related to Figure 2:

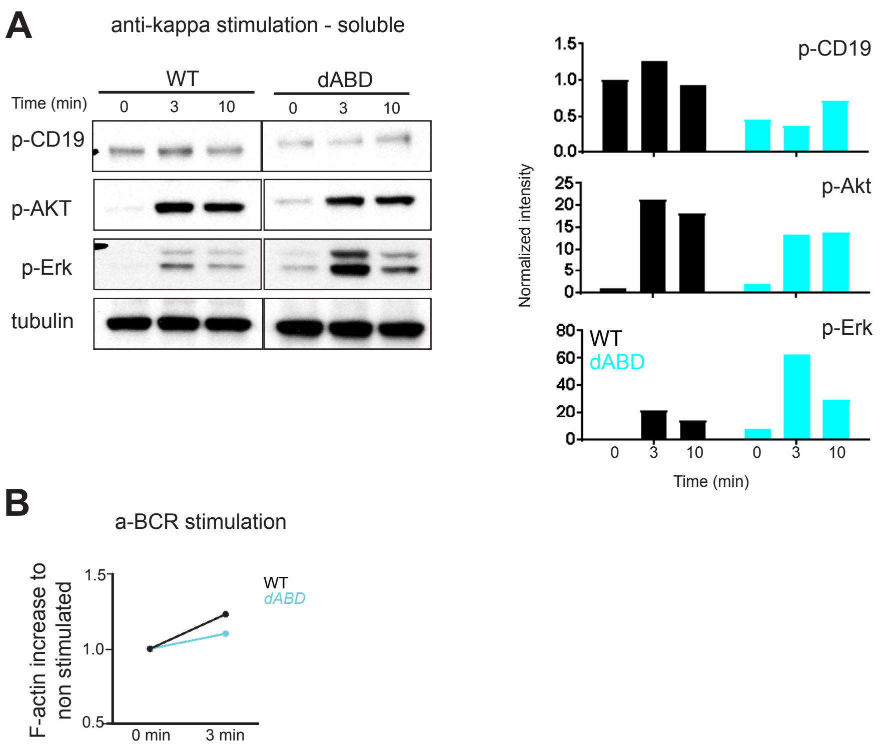

Figure S2 – related to Figure 2:

(A) Immunoblot of splenic WT or WIPΔABD B cells stimulated with soluble anti-κ chain antibody and probed with antibodies as indicated. Quantifications of intensity of proteins normalized by densiometry to tubulin and to the signal in unstimulated WT cells at t = 0. Data are representative of at least two independent experiments.

(B) F-actin increase indicating the polymerisation of F-actin measured by flow cytometry. Purified splenic WT or WIPΔABD B cells were stimulated with soluble anti-κ chain antibody for 3 min, fixed and stained with phalloidin. Graph indicates the increase in the geometric mean-fluorescent intensity (gMFI) normalised to the gMFI of phalloidin staining in the respective unstimulated cells. Data are representative of at least two independent experiments.

Figure S3 - related to Figure 5:

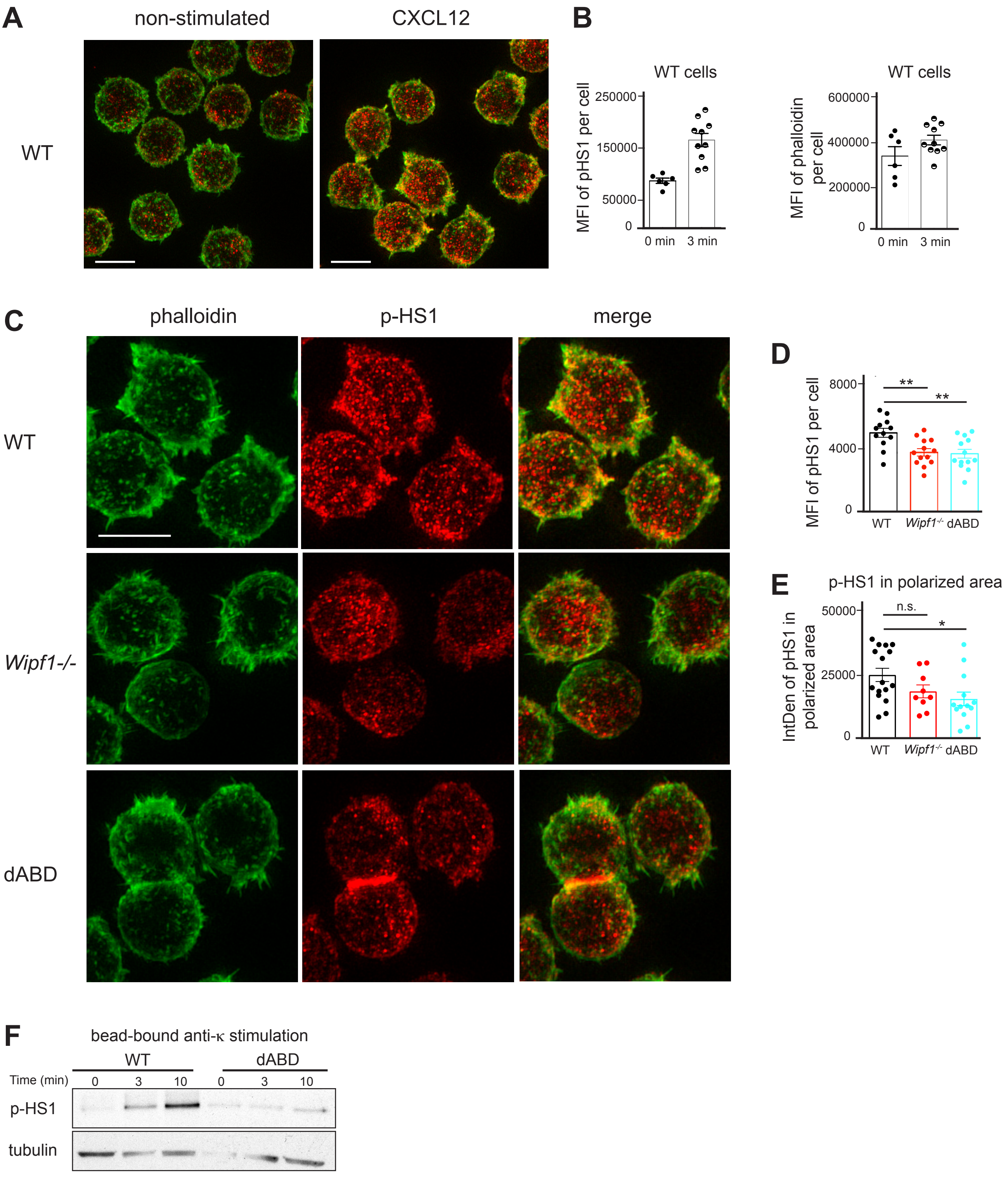

Figure S3 – related to Figure 5:

(A) Representative SIM images (Maximum Intensity projections) of phalloidin (green) and p-HS1 (red) staining in fixed WT B cells settled on coverslips coated with poly-L-lysin and non-stimulated or stimulated with CXCL12 (200ng/ml) for 3 min. Scale bar, 5 μm.

(B) Quantifications show the mean fluorescence intensity (MFI) of p-HS1 (left) or phalloidin (right) per cell as determined by ImageJ before and after 3 minutes of stimulation of WT B cells.

(C) Representative SIM images (Maximum Intensity projections) of phalloidin (green) and p-HS1 (red) staining in B cells settled on coverslips coated with poly-L-lysin and treated with CXCL12 (200ng/ml) for 3 min. Scale bar, 5 μm.

(D) Quantifications show MFI of p-HS1 per cell as analysed by the ImageJ software after stimulation of WT, *Wipf1*$^{-/-}$ and WIPΔABD B cells.

(E) Quantification of the fluorescence intensity of p-HS1 in the polarised area per cell as analysed by the ImageJ software. Each dot represents a cell. About 100 cells were analysed per genotype. Data are representative of at least 2 independent experiments.

(F) Immunoblot of splenic WT and WIPΔABD B cells treated with bead-bound anti-k chain antibodies and probed with antibodies against p-HS1 and tubulin. This experiment and experiment in Figure 2A were performed simultaneously. The loading control measurements were part of both experiments. Data are representative of two independent experiments.

B cell function dependent on	WIP	WIP binding to actin
	Survival, differentiation, migration, chemotaxis	Migration, chemotaxis to CXCL12
	GC formation, antibody production	Antibody affinity maturation
	CD19 mobility, CD19 phosphorylation, PI3K signaling	CD19 phosphorylation, PI3K signaling (partially)
	Actin stabilization (foci formation)	Actin stabilization (foci formation partially)<br>HS1 localization

Table S1: comparison of B cell functions dependent on WIP or WIP binding to actin – related to Figures 1-5
